# Coenzyme Q deficiency may predispose to sudden unexplained death via an increased risk of cardiac arrhythmia

**DOI:** 10.1007/s00414-024-03265-5

**Published:** 2024-06-07

**Authors:** Shouyu Wang, Cordula Haas, Zhimin Wang, Jianghua Du, Zijie Lin, Guanghui Hong, Liliang Li, Ruiyang Tao, Yiwen Shen, Jacqueline Neubauer

**Affiliations:** 1 Department of Forensic Medicine, School of Basic Medical Sciences, Fudan University, Shanghai, 200032 China; 2 Zurich Institute of Forensic Medicine, University of Zurich, Zurich, 8057 Switzerland; 3 Shanghai Key Laboratory of Forensic Medicine, Shanghai Forensic Service Platform, Academy of Forensic Sciences, Ministry of Justice, Shanghai, 200063 China

**Keywords:** Sudden unexplained death, Postmortem molecular autopsy, Genetic burden, Cardiac arrhythmia, Coenzyme Q deficiency

## Abstract

Cardiac arrhythmia is currently considered to be the direct cause of death in a majority of sudden unexplained death (SUD) cases, yet the genetic predisposition and corresponding endophenotypes contributing to SUD remain incompletely understood. In this study, we aimed to investigate the involvement of Coenzyme Q (CoQ) deficiency in SUD. First, we re-analyzed the exome sequencing data of 45 SUD and 151 sudden infant death syndrome (SIDS) cases from our previous studies, focusing on previously overlooked genetic variants in 44 human CoQ deficiency-related genes. A considerable proportion of the SUD (38%) and SIDS (37%) cases were found to harbor rare variants with likely functional effects. Subsequent burden testing, including all rare exonic and untranslated region variants identified in our case cohorts, further confirmed the existence of significant genetic burden. Based on the genetic findings, the influence of CoQ deficiency on electrophysiological and morphological properties was further examined in a mouse model. A significantly prolonged PR interval and an increased occurrence of atrioventricular block were observed in the 4-nitrobenzoate induced CoQ deficiency mouse group, suggesting that CoQ deficiency may predispose individuals to sudden death through an increased risk of cardiac arrhythmia. Overall, our findings suggest that CoQ deficiency-related genes should also be considered in the molecular autopsy of SUD.

**Supplementary Information:**

The online version contains supplementary material available at 10.1007/s00414-024-03265-5.

## Introduction

The etiology of unexpected sudden death is diverse and highly correlated with age of onset [1]. Coronary artery disease is thought to be the major cause of unexpected sudden death in older people [2]. However, in younger populations, it often strikes apparently healthy children and young adults without prior clinical symptoms or diagnosed diseases [3, 4]. In up to one third of these deceased, no clear cause of death can be established, even after a comprehensive medico-legal investigation, including pathological/toxicological assessment and forensic investigation of the circumstances of death [5, 6]. In such cases, the deaths could be termed as sudden infant death syndrome (SIDS) in an individual younger than one year of age [7], sudden unexplained death (SUD) in an individual older than 1 year of age, or sudden arrhythmic death syndrome (SADS) for cases with a negative pathological and toxicological assessment [8].

Understanding the exact causes of SUD will not only improve the accuracy of pathological diagnosis, but also provide important information about the genetic background of rare cardiovascular diseases. Although the pathophysiological mechanisms of SUD remain incompletely understood, an increasing number of studies have shown that cardiac arrhythmia could be the direct cause of death in the majority of SUD cases. Genetic testing and functional assays have identified numerous pathogenic single nucleotide variants (SNVs) or structural variations (SVs) located in genes associated with primary arrhythmia syndromes [9]. However, there is still a large proportion of SUD cases that remain unexplained even after post-mortem molecular autopsy [10, 11]. It is therefore very likely that the genetic predisposition and corresponding endophenotypes contributing to SUD are more diverse than expected.

Coenzyme Q (CoQ) is a lipophilic molecule synthesized in the mitochondria. It consists of a quinone group and of a polyisoprenoid tail of variable length in different species: yeast has six units (CoQ6), mice nine (CoQ9) and humans ten (CoQ10) [12]. A central role of CoQ is to act as an electron transporter in mitochondrial respiratory chain (MRC) function [13]. CoQ deficiency is a rare mitochondrial disorder associated with heterogeneous phenotypes and a characteristic decrease in CoQ10 levels in human tissues and plasma [14]. In general, CoQ deficiency due to variants in genes encoding proteins of the CoQ biosynthesis pathway or its regulation is defined as primary CoQ deficiency. When CoQ deficiency is caused by other metabolic disorders or genetic defects unrelated to the CoQ biosynthesis pathway, it is referred to as secondary CoQ deficiency [15]. Both primary and secondary CoQ deficiencies can lead to varying degrees of clinical manifestations, with single or multiple organ systems involved.

Sufficient CoQ is essential for normal heart function, as cardiac contraction and activation of ATP-regulated membrane channels depend on energy supply from MRC function. Previous studies have shown that circulating CoQ levels are significantly lower in patients with dilated cardiomyopathy compared to healthy controls [16]. However, it remains unclear whether CoQ deficiency contributes to arrhythmic death. When investigating the genetic predisposition to SUD, previous studies have mainly focused on known susceptibility genes for ion channelopathies, inherited cardiomyopathy or epilepsy, while the involvement of deleterious variants in CoQ deficiency-related genes has always been overlooked [17–19]. To comprehensively evaluate the role of CoQ deficiency in SUD, we re-analyzed the available exome sequencing data of our SUD and SIDS cohorts [20, 21], focusing on the CoQ deficiency-related genes that have not been investigated before. In addition, the correlation observed at the genetic level was examined in a mouse model through electrophysiological and morphological experiments, to test whether CoQ deficiency could predispose to SUD.

## Materials and methods

### Exome sequencing data re-analysis

Initially, 45 SUD cases and 155 SIDS cases from our previous studies were re-evaluated for the purpose of this research. The inclusion criteria, autopsy findings and sequencing procedures have been described previously [20, 21]. The SUD cohort consisted of 45 cases with a mean age (± SD) of 30.2 (± 14.5) years (range: 1–63 years of age) and a mean body mass index (± SD) of 24.9 (± 4.9) (range: 13.7–35.5). 34 (76%) of the deceased were male and the majority were of European origin (89%). As the exome sequencing data of 4 SIDS cases were not available for re-analysis, the SIDS dataset consisted of 151 exomes. The deceased SIDS infants had a mean age (± SD) of 17.6 (± 10.7) weeks (range: 0.6–48.1 weeks), 91 (60%) were boys and all of them were of European origin.

Genetic investigation was confined to a target gene panel consisting of 44 known human CoQ deficiency-related genes (Geneset B) [22]. An overview of the genes and their constraint metrics [23] are shown in Supplementary Table S1. Most of the CoQ deficiency-related genes have not been previously analyzed in the SUD study (Geneset A: consisted of 244 genes [20]) and the SIDS study (Geneset C: consisted of 192 genes [21]), with the exception of 7 genes (*ACADVL*, *BRAF*, *ETFB*, *ETFDH*, *FXN*, *MT-TL1*, and *PDSS2*) that have been reported to participate in cardiovascular/metabolic diseases. Variant filtering was performed according to the following rules: (1) functional impact-based filtering retaining only exonic variants that are non-synonymous, frameshift, and nonsense, as well as splice-site and untranslated region (UTR) variants; (2) allele frequency-based filtering retaining only variants with a minor allele frequency (MAF) < 0.1% in all populations according to the Genome Aggregation Database (gnomAD) [24]. Subsequently, an automated pathogenicity assessment and variant classification were performed according to the ACMG/AMP guidelines using Varsome v.10.1 [25, 26].

### Gene-based burden testing

To further compare the aggregate burden of rare variants in the 44 CoQ deficiency-related genes between our case cohorts and the general population, without considering their predicted functional effects, a burden test including all rare exonic and UTR variants identified in the previous step was performed using the software package TRAPD and 60,706 Exome Aggregation Consortium (ExAC) data sets from gnomAD [27]. Compared to the commonly used single-variant-based tests [28], a gene-based burden test, which assesses the cumulative effect of multiple variants influencing the phenotype in the same direction, could improve the power to detect statistical signals between case and control subjects. Based on the number of case and control subjects carrying and not carrying a qualifying variant in each gene, P values were calculated using the two-sided Fisher’s exact test. For the dominant model, the number of cases carrying at least one qualifying variant in each gene was tabulated. For the recessive model, the case cohort counts were generated by tabulating the number of cases carrying two or more qualifying variants in each gene. P values < 0.05 were considered significant for both models. For a better illustration, quantile-quantile plots of the initial burden test results were generated.

### CoQ deficiency mouse model

The CoQ deficiency mouse model was induced using 4-nitrobenzoate (4-NB) [29]. Briefly, 3-week-old C57Bl/6 mice (9 males and 9 females for each group) were treated with 0.05 M 4-NB solution by oral administration for 6 weeks. Soluble 4-NB was prepared by adding 8.36 mg/ml 4-nitrobenzoic acid (Merck KGaA, Darmstadt, Germany) and 4.21 mg/ml sodium bicarbonate (Merck KGaA, Darmstadt, Germany) to the sterile drinking water. Since sodium bicarbonate, which serves to increase the solubility, could be an additional confounding factor, a sex-matched control group A (Ctrl-A) treated with 0.05 M sodium bicarbonate solution was used. In addition, a blank control group B (Ctrl-B) was recruited without any treatment.

### Electrophysiological and morphological measurements

Mice were anaesthetized with 1–3% isoflurane in oxygen and kept warm with a heating pad. Surgical steel needle electrodes were then placed under the skin of each limb and the electrocardiogram (ECG) was recorded in lead I and lead II configuration. Baseline ECGs were recorded for approximately 10 min until a stable baseline and heart rate (HR) were achieved. Transthoracic Doppler echocardiograms were measured using the Vinno 6 platform (VINNO Technology, Suzhou, China). M-mode cine loops were acquired in 2-dimensional parasternal short-axis view at the papillary muscle level to measure interventricular septal end-diastole and end-systole (IVSd and IVSs), left ventricular internal diameter end diastole and end systole (LVIDd and LVIDs), left ventricular posterior wall end-diastole and end-systole (LVPWd and LVPWs), left ventricular end-diastolic and end-systolic volume (LVEDV and LVESV), ejection fraction (EF) and fractional shortening (FS) at the time of echocardiography. All measurements were performed in triplicate. Mice were sacrificed and autopsied immediately after the experiments for body and heart weight measurements.

### CoQ extraction and quantification

Weighed heart tissue samples were homogenized after mixing with 500 µL methyl tert-butyl ether (MTBE), 100 µL methanol, and 250 µL water. After centrifugation at 12,000 rpm for 15 min, a 300 µL aliquot of the MTBE layer was transferred to a new tube and dried under a gentle nitrogen flow. The residue was reconstituted with 300 µL of 50% acetonitrile/2-propanol and centrifuged at 12,000 rpm for 15 min. A 10 µL aliquot of each sample was then mixed with 290 µL of 50% acetonitrile/2-propanol containing CoQ10-d6 internal standard (Cayman Chemical, Michigan, USA) and transferred to an autosampler vial for UHPLC-MRM-MS analysis. A series of calibration standard solutions were prepared by stepwise dilution of the CoQ9 standard stock solution (Merck KGaA, Darmstadt, Germany) to generate a calibration curve, and the least squares method was used for regression fitting. An Agilent 1290 Infinity II series UHPLC System and an Agilent 6460 triple quadrupole mass spectrometer were applied for UHPLC separation and assay development.

### Statistical analysis

Statistical analysis was performed with GraphPad Prism software v 8.3.0 (La Giolla, CA, USA). Continuous variables, such as PR interval, were presented as mean ± standard error. Differences between groups were compared using the one-way ANOVA test for normally distributed continuous variables with homogeneous variance, otherwise the Kruskal-Wallis test was used. All statistical tests were two-sided and a P value < 0.05 was considered statistically significant.

## Results

### Variants with likely functional effects in CoQ deficiency-related genes

**Fig. 1:**
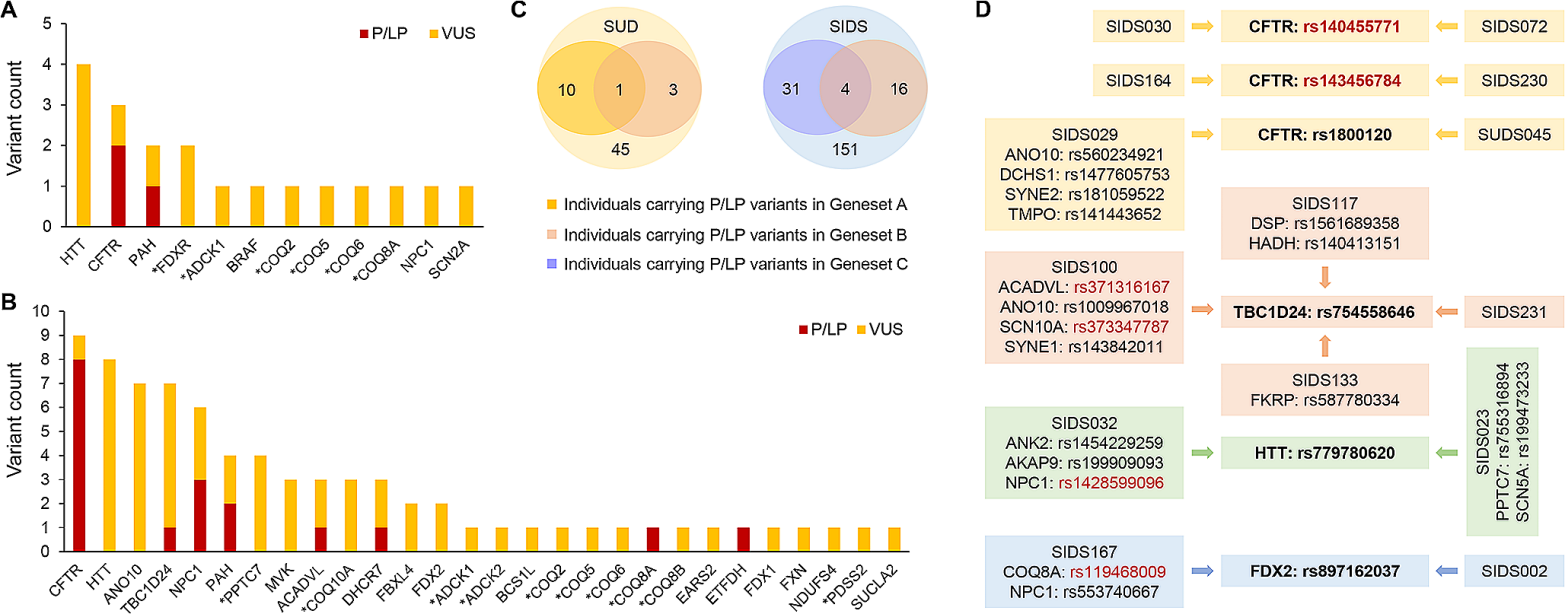
Rare variants with likely functional effects in the case cohorts. Genes marked with an asterisk indicate primary CoQ deficiency-related genes. **(A)** 19 rare P/LP variants or VUS distributed in 12 known human CoQ deficiency-related genes were identified in 17 out of the 45 SUD cases (38%). **(B)** 76 rare P/LP variants or VUS distributed among 28 known human CoQ deficiency-related genes were identified in 56 out of the 151 SIDS cases (37%). **(C)** P/LP variants in genes associated with cardiovascular/metabolic diseases were previously identified in 10 SUD cases and 31 SIDS cases, respectively. Among the 3 SUD cases and 16 SIDS cases harboring at least one of the P/LP variants in Geneset B (44 human CoQ deficiency-related genes), P/LP variants in Geneset A (244 genes analyzed in the SUD study) and Geneset C (192 genes analyzed in the SIDS study) were also identified in 1 SUD case and 4 SIDS cases, respectively. **(D)** Among the P/LP variants or VUS identified, 6 variants occurred in two or more cases (highlighted in bold). These included 2 LP variants (rs140455771 and rs143456784) and 4 VUS (rs1800120, rs754558646, rs779780620, and rs897162037). The 4 VUS were also found to coexist with several other P/LP variants (highlighted in red) or VUS in genes associated with cardiovascular/metabolic diseases or CoQ deficiency

A total of 3 rare pathogenic or likely pathogenic (P/LP) variants were found in 3 different SUD cases (7%). Two of these variants were identified in the exonic region of *CFTR* and one in the exonic region of *PAH*. In addition, 16 variants of uncertain significance (VUS) in 12 genes were identified in 14 (31%) SUD cases. Among these VUS, 10 were located in the exonic region and 6 in the UTR. In total, 17 (38%) SUD cases were identified as harboring rare variants with likely functional effects (Supplementary Table S2). A total of 7 and 12 rare variants with likely functional effects were found to affect primary CoQ deficiency-related genes and secondary CoQ deficiency-related genes, respectively. Among the affected genes, *HTT* has the highest prevalence of variants in the SUD cohort (Fig. 1A). In terms of clinical evidence, 12 (63%) of the variants were not evaluated before, since an interpretation for these variants in the ClinVar database was not available. Additional demographic characteristics of the above mentioned 17 SUD cases can be found in Supplementary Table S3.

In the SIDS cohort, 18 rare P/LP variants were identified in 8 genes (*ACADVL*, *CFTR*, *COQ8A*, *DHCR7*, *ETFDH*, *NPC1*, *PAH*, and *TBC1D24*) in 16 cases (11%), while another 58 rare VUS were identified in 26 genes in 46 (30%) cases. Taken together, a total of 56 (37%) SIDS cases were found to harbor rare variants with likely functional effects. Of the total 76 rare variants with likely functional effects, 40 were located in the exonic region and 36 in the UTR (Supplementary Table S4). As a secondary CoQ deficiency-related gene, *CFTR* has the highest prevalence of variants in the SIDS cohort (Fig. 1B). In terms of clinical evidence, 40 (53%) of the variants were novel and thus not evaluated in the ClinVar database before.

Within the 3 SUD cases and the 16 SIDS cases harboring at least one of the P/LP variants in the 44 CoQ deficiency-related genes, we had previously identified several P/LP variants in cardiovascular/metabolic disease-associated genes. Since 37 out of the 44 CoQ deficiency-related genes (Geneset B) were not included in either Geneset A or Geneset C, carriers with shared variants from different genesets were further investigated. As shown in Fig. 1C, one of the SUD cases (SUDS112) was found to harbor P/LP variants in the two Genesets A and B. Specifically, one LP variant was detected in Geneset B (*CFTR*) and 3 P/LP variants in Geneset A (*ANK2*, *CALR3*, and *MLYCD*) (Supplementary Table S5). In addition, 4 SIDS cases (SIDS045, SIDS091, SIDS100, and SIDS156) were found to harbor P/LP variants in the two Genesets B and C (Fig. 1C), with a total of 7 P/LP variants in Geneset B (*ACADVL, ANO10, CFTR, DHCR7*, and *TBC1D24*) and a total of 5 P/LP variants in Geneset C (*ACADVL, CASQ2*, *SCN1B*, *SCN10A*, and *SYNE1*) (Supplementary Table S5).

Among the P/LP variants or VUS identified in our SUD and SIDS cohorts, 6 variants appeared in two or more cases (Fig. 1D and Supplementary Table S6). One of the LP variants, *CFTR*: rs140455771, was the only variant identified in SIDS030 and SIDS072 after a thorough review of genes associated with cardiovascular/metabolic diseases and CoQ deficiency, suggesting that this variant might be a finding that deserves further functional investigation. Similarly, another LP variant, *CFTR*: rs143456784, was the only positive finding in SIDS164 and SIDS230. In addition, the 4 VUS identified in this study (*CFTR*: rs1800120, *TBC1D24*: rs754558646, *HTT*: rs779780620, and *FDX2*: rs897162037) were found to coexist with several other P/LP variants or VUS in genes associated with cardiovascular/metabolic diseases or CoQ deficiency. Thus, it was not possible to associate the phenotypic information of these cases with specific variants due to their complex genetic background.

### Burden testing of rare exonic and UTR variants

**Fig. 2:**
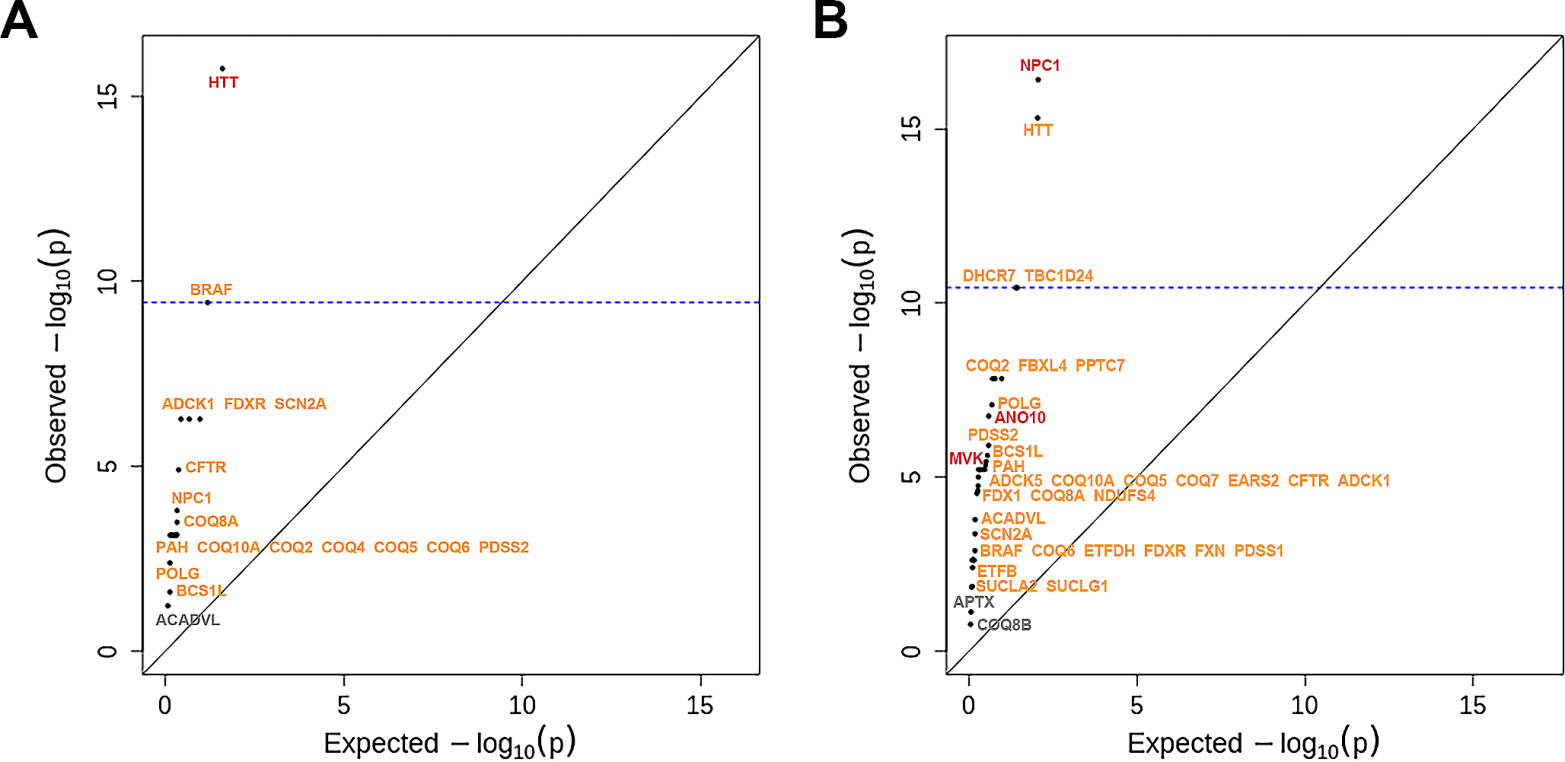
Burden testing of rare exonic and UTR variants identified in human CoQ deficiency-related genes. The x-axis represents the expected –log_10_(P) under a uniform distribution of p-values. The y-axis shows the observed –log_10_(P) from the burden testing data. Each data point represents a single gene. Genes highlighted in red indicate that there is a significant burden under both the dominant and the recessive models. Genes highlighted in orange indicate that there is a significant burden under the dominant model only. Genes in gray indicate that there is no significant burden under either model. **(A)** Significant genetic burden was observed in 17 genes (16 dominant and one both) in the SUD cohort. **(B)** Significant genetic burden was observed in 34 genes (31 dominant and 3 both) in the SIDS cohort

A gene-based burden test between the SUD cases and 60,706 controls from the gnomAD datasets revealed a significant genetic burden in 17 out of the 18 genes containing at least one rare exonic/UTR variant under the dominant model, indicating a strong association of these genes with SUD in a dominant state. Besides, *HTT* also reached strong association signals under the recessive model, suggesting that variants in this gene might be pathogenic even in a recessive state (Fig. 2A and Supplementary Table S7). For SIDS cases, a significant genetic burden was observed in 34 out of the 36 genes containing at least one rare exonic/UTR variant under the dominant model. Among these, 3 genes (*ANO10*, *MVK*, and *NPC1*) also reached strong association signals under the recessive model (Fig. 2B and Supplementary Table S8). In general, the significant burden identified in the majority of CoQ deficiency-related genes confirmed that these genes harbor an excess of rare exonic and UTR variants in the SUD and SIDS cohorts compared to the general population.

### Abnormal ECG patterns

**Fig. 3:**
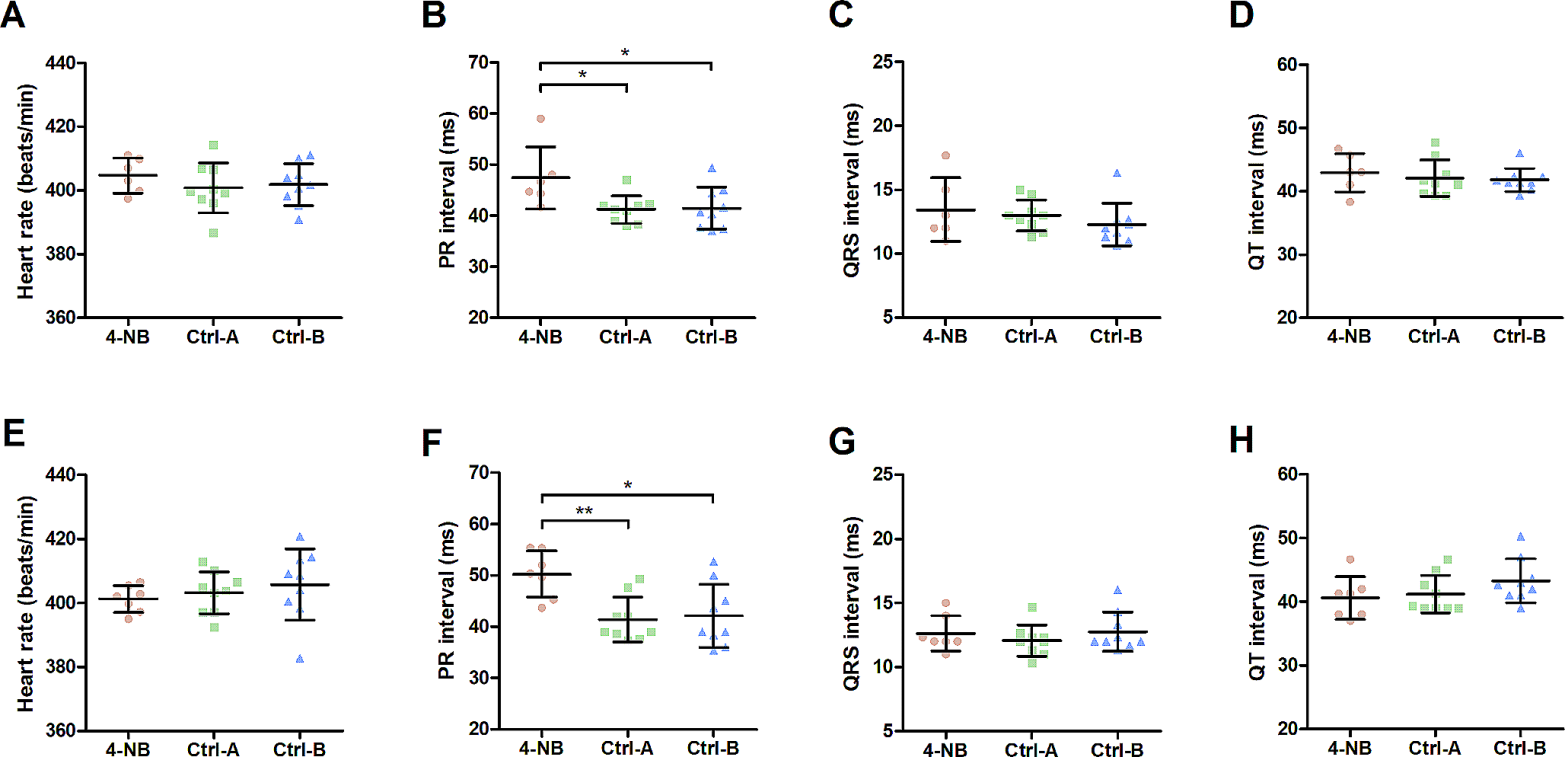
Comparative analysis of electrocardiographic observations in the 4-NB and control groups. Each data point represents the average value of 3 replicates for the same mice. For both males **(A-D)** and females **(E-H)**, QRS and QT intervals were similar between groups at steady heart rate, whereas PR interval was significantly longer in the 4-NB group (N _male_ = 6; N _female_ = 7) compared to Ctrl-A (N _both_ = 9) and Ctrl-B (N _both_ = 9) (*P < 0.05; **P < 0.005)

**Fig. 4:**
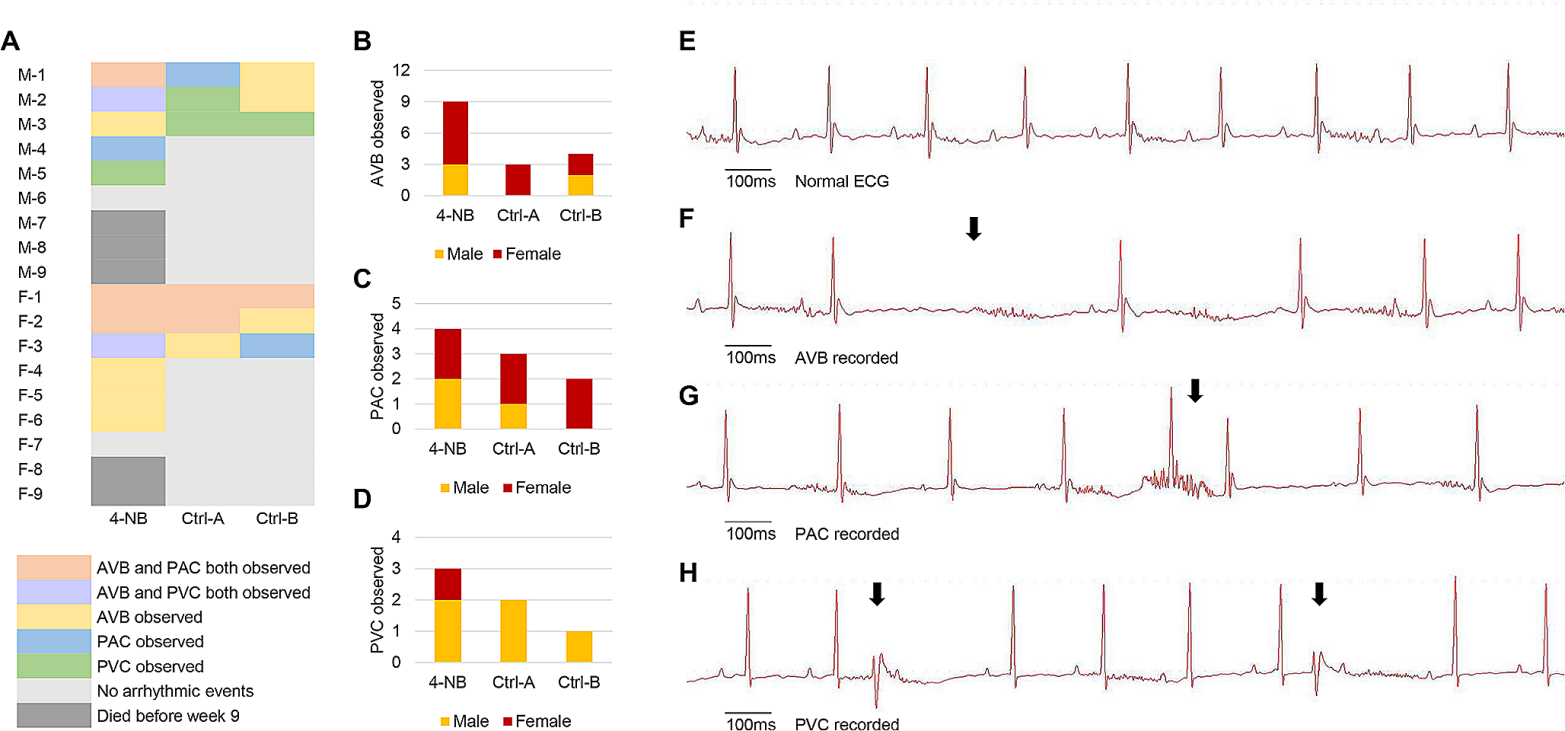
Differences in the occurrence of arrhythmic events and representative surface ECG recordings. **(A)** Colored cells indicate different scenarios where certain types of arrhythmic events were observed for at least once during the measurements. **(B-D)** The total number of subjects with different arrhythmic events observed. **(E-H)** The illustration of normal ECG recordings versus typical AVB, PAC, and PVC

A total of 3 male and 2 female mice from the 4-NB group died unexpectedly during the night between week 6 and week 7. For the remaining mice, electrophysiological and morphological measurements were performed during week 9. To investigate the effect of CoQ deficiency on cardiac electrical activity, the ECG and echocardiographic patterns of the 4-NB and control groups were compared under equivalent HR conditions. ECG analysis showed that most parameters were not affected by the decrease in myocardial CoQ concentration. However, the PR interval was significantly prolonged in the 4-NB group compared with the control groups, irrespective of sex (Fig. 3). Moreover, during the same observation period, the 4-NB group developed more frequent isolated arrhythmic events as compared to the Ctrl-B group, including premature ventricular contraction (PVC), premature atrial contraction (PAC) and atrioventricular block (AVB) (Fig. 4).

### Morphological and histological characteristics

Overall, the body weight increase of the 4-NB group was slower than that of the control groups throughout the growth phase. Significant differences in body weight and heart weight were observed between the 4-NB and control groups at week 9. However, the heart weight to body weight ratio (HW/BW) remained unchanged after 6 weeks of treatment (Supplementary Figure S1). In terms of echocardiographic patterns, the IVS, LVID and LVPW thickness were generally reduced in the 4-NB group. The difference was particularly evident in female mice, as LVPW at end systole was the only parameter with a statistically significant difference between groups (Table 1). Nevertheless, no difference in EF and FS was observed.

**Table 1:** Echocardiographic measurements in the 4-NB and control groups

Parameters	Male			Female		
	4-NB (*N* = 6)	Ctrl-A (*N* = 9)	Ctrl-B (*N* = 9)	4-NB (*N* = 7)	Ctrl-A (*N* = 9)	Ctrl-B (*N* = 9)
IVSd (mm)	0.39 ± 0.07	0.41 ± 0.03	0.46 ± 0.06	0.36 ± 0.04	0.39 ± 0.03	0.38 ± 0.04
IVSs (mm)	0.55 ± 0.09	0.62 ± 0.07	0.63 ± 0.10	0.52 ± 0.04	0.60 ± 0.08	0.55 ± 0.08
LVIDd (mm)	3.75 ± 0.40	4.13 ± 0.26	4.04 ± 0.53	3.76 ± 0.25	3.87 ± 0.31	3.79 ± 0.26
LVIDs (mm)	2.82 ± 0.43	3.07 ± 0.29	3.00 ± 0.52	2.86 ± 0.19	2.92 ± 0.30	2.76 ± 0.16
LVPWd (mm)	0.46 ± 0.06	0.5 ± 0.10	0.55 ± 0.09	0.45 ± 0.09	0.47 ± 0.03	0.5 ± 0.05
LVPWs (mm)	0.65 ± 0.10	0.77 ± 0.09	0.78 ± 0.14	0.57 ± 0.08 *	0.66 ± 0.06	0.72 ± 0.12
LVEDV (ml)	0.14 ± 0.04	0.18 ± 0.03	0.17 ± 0.06	0.14 ± 0.03	0.15 ± 0.03	0.14 ± 0.03
LVESV (ml)	0.06 ± 0.03	0.08 ± 0.02	0.08 ± 0.04	0.06 ± 0.01	0.07 ± 0.02	0.06 ± 0.01
EF (%)	56.15 ± 8.00	57.05 ± 6.99	57.46 ± 7.13	54.48 ± 1.55	55.67 ± 5.35	59.41 ± 4.49
FS (%)	25.16 ± 4.73	25.70 ± 3.94	25.94 ± 4.06	23.92 ± 0.90	24.75 ± 3.19	26.97 ± 2.77
HR	391.68 ± 12.24	427.42 ± 59.87	405.17 ± 54.3	399.58 ± 37.79	425.2 ± 54.15	390.47 ± 61.41

* Compared with Ctrl-B (P<0.05)

IVSd and IVSs, interventricular septal end diastole and end systole; LVIDd and LVIDs, left ventricular internal diameter end diastole and end systole; LVPWd and LVPWs, left ventricular posterior wall end diastole and end systole; LVEDV and LVESV, left ventricular end diastolic and end systolic volume; EF, ejection fraction; FS, fractional shortening; HR, heart rate at the time of echocardiography

In general, no obvious structural defects of the heart or other organs were observed at autopsy. To assess the degree of fibrosis in the different groups, the histological characteristics of the mouse heart sections were further examined using Masson’s trichrome staining. As shown in representative histological images (Supplementary Figure S2A-C), no significant cardiomyocyte changes were observed in the 4-NB group. CoQ quantification of frozen heart tissues was then performed to validate whether the CoQ deficiency model was successfully constructed. The correlation coefficient (R^2^) of the regression fitting was 0.9991 for CoQ9, indicating a satisfactory quantitative relationship between MS response and CoQ9 concentration. As expected, the myocardial CoQ9 concentration was significantly lower in the 4-NB group compared to the control groups (Supplementary Figure S2D-E).

## Discussion

Due to the essential role of CoQ in all energy-dependent processes of the heart, CoQ deficiency is known to be involved in various cardiac diseases [16]. However, the association between CoQ deficiency and SUD has not been investigated. Here, we present evidence from both the genetic level and physiological levels to support that CoQ deficiency is a previously overlooked predisposing factor for SUD. Re-evaluation of our exome sequencing data revealed that both the SUD and SIDS cohorts carried a substantial number of rare variants with likely functional effects in the 44 CoQ deficiency-related genes. Although pathogenic or conflicting clinical evidence has been previously reported for some of these variants, more than half of the variants identified in either the SUD or SIDS cohorts remain unexplored. Combined with the results from the burden tests and the fact that all of these variants were ultra-rare (MAF < 0.1% in gnomAD), it is highly likely that a substantial proportion of them are also clinically relevant for different CoQ deficiency phenotypes. However, since the clinical symptoms of mild CoQ deficiency are very often non-specific and CoQ is not chemically stable [14], it can be difficult to determine whether the variant carriers were suffering from CoQ deficiency by content assay of human CoQ10 in forensic samples.

In terms of cardiac electrical activity, the 4-NB induced CoQ deficiency mouse group had a significantly prolonged PR interval and a higher incidence of arrhythmic events, suggesting that CoQ deficiency is an independent pathophysiological condition that promotes cardiac arrhythmia. Furthermore, the incidence of AVB in the 4-NB group was more than twice that of the two control groups, even though the sample size of the 4-NB group was smaller. Since the AVB events recorded in our study were mostly accompanied by PR prolongation, these observations are most likely to be vagally-mediated AVB rather than paroxysmal AVB [30]. Although vagally-mediated AVB could lead to neutrally-mediated syncope, it is generally considered to be benign. Nevertheless, due to its poor recognition and unpredictability, its prevalence and consequences might be underestimated, especially in apparently healthy young adults [31]. Our findings also highlight the need for further evaluation of the prognostic significance of vagally-mediated AVB. Another important finding of our study is that 3 male mice and 2 female mice of the 4-NB group died at night during week 6 to 7 without any apparent injury. However, since no ECG or symptoms were recorded prior to death, we were unable to determine whether the cause of death was cardiac arrhythmia. According to previous reports, vagally-mediated AVB appears to be more common at night [32], which could be due to an acute increase in vagal tone during sleep. In a recent epidemiological study, Ramireddy et al. reported that female sex and central nervous system-affecting medications were independently associated with nocturnal sudden cardiac death [33]. However, since humans and mice have completely reversed circadian rhythms, there might be little correlation between the death in our 4-NB model and increased vagal tone.

According to previous studies, CoQ deficiency is common in cardiomyopathy and has been reported to be associated with chronic heart failure [16, 34]. However, no indicative echocardiographic features or histological findings were identified in our 4-NB group. This is most likely because our intervention period is too short for obvious morphological changes. Instead, we observed a significant effect of 6 weeks of 4-NB treatment on overall growth rate, resulting in reduced body and heart size and weight. Growth retardation is a commonly observed phenotype in patients with primary CoQ deficiency [35]. Combined with the finding that myocardial CoQ9 concentration was significantly lower in the 4-NB group compared to the control groups, we considered the abnormal growth curves of 4-NB treated mice further confirm that our CoQ deficiency model was able to mimic primary CoQ deficiency in its early stage.

As a typical polygenic disease, both primary and secondary CoQ deficiencies have shown a wide clinical variability, including the age of onset, the severity of phenotype and the degree of CoQ reduction in tissues [36]. Since secondary CoQ deficiency-related genes are not involved in CoQ biosynthesis, the functional effects of P/LP variants in these genes remain unclear. As a secondary CoQ deficiency-related gene and a well-known disease-causing gene for the Huntington’s disease, *HTT* has recently been associated with progressive cardiac arrhythmia and ECG abnormalities [37]. Although the underlying mechanism of cardiac involvement in Huntington’s disease is not fully understood, these observations suggest that *HTT* dysfunction is an independent predisposing factor for cardiac arrhythmia and SUD, whether accompanied by CoQ deficiency or not. Similarly, for the other secondary CoQ deficiency-related genes identified in this study, their actual role in SUD and the underlying pathogenic mechanisms deserve further investigation.

In the SUD and SIDS cases where we have identified genetic variants predisposing to CoQ deficiency, a small proportion of P/LP variants or VUS are also present in genes associated with cardiovascular/metabolic diseases [20, 21]. Although the identification of P/LP variants in certain genes is usually considered strong pathogenic evidence [10, 11], there is still no consensus on VUS due to the conflicting interpretations of their deleteriousness [38]. As the number of SUD susceptibility genes increases, a possible explanation for the phenotypic severity difference observed in VUS carriers is that there may be a joint effect of multiple genetic variants. Based on the multifactorial model of SUD [39, 40], there may be multiple genetic variants contributing to lethal arrhythmia. The effect of a single VUS on cardiac function might be limited. However, once combined with other intrinsic or extrinsic predisposing factors (for example, alcohol intake or physical exercise), the threshold for irreversible consequences could be reached. Based on our findings, genetic variants predisposing to CoQ deficiency could be one such intrinsic factor that increases the risk of SUD.

Currently, the interpretation of molecular autopsy findings in SUD still follows the logic of the management of monogenic diseases. Thus, the genetic investigation of SUD is mainly focused on very limited variants with the most prominent functional effects (e.g. P/LP variants in ion channel-encoding genes). However, a polygenic risk score that allows the estimation of combined SUD risk from multiple genetic variants could be developed, similar to the recently proposed strategy for arrhythmia risk assessment [41]. In future studies, efforts should be made to develop such a score, in order to accurately predict individualized SUD risk in suspected deceased. Although our work provided only preliminary evidence that CoQ deficiency may predispose to SUD via an increased risk of cardiac arrhythmia, the genetic and physiological findings suggest that CoQ deficiency-related genes should also be considered in the molecular autopsy of SUD. However, as the dysfunction of these genes might not have the same magnitude of effect as the dysfunction of ion channel-encoding genes, appropriate weights of different genes should be included.

It is also worth noting that one of the LP variants (*ACADVL*: rs371316167) identified in SIDS100 was previously reported in our study [21], as *ACADVL* is also a known susceptibility gene for SUD and SIDS. However, this variant was previously classified as VUS. The change of pathogenicity classification in patients with genetic diseases is frequently observed in clinical practice [42], largely due to the rapid emergence of new evidence provided by ongoing genotype-phenotype correlation studies, constantly updated population databases, and rapidly iterated prediction algorithms. Therefore, periodic re-evaluation of rare variants originally classified as ambiguous is encouraged [43], as it might provide the forensic experts with new evidence to explain the previously unexplained death.

Our current study has several limitations. First, we were not able to clarify whether the observed difference in the number of variants between the SUD and SIDS cohorts was simply due to sampling error, or whether there are actually different genetic backgrounds among unexplained death cases at different ages. Besides, our CoQ deficiency mouse model was drug-induced, which might not accurately simulate real patients with endogenously reduced CoQ levels caused by genetic defects. In future studies, telemetry ECG measurements could be performed on a conditional gene knockout mouse model to obtain more comprehensive electrophysiological properties. In addition, it is important for subsequent studies to independently validate the actual contribution of different CoQ deficiency-related genes to SUD through multi-center studies and functional assays.

## Conclusion

In conclusion, we observed a significant genetic burden of human CoQ deficiency-related genes in the SUD and SIDS cohorts. Further electrophysiological and morphological investigation revealed a significantly prolonged PR interval and an increased incidence of AVB in the 4-NB-induced CoQ deficiency mouse model, independent of sex. Our results suggest that CoQ deficiency may predispose to sudden death via an increased risk of cardiac arrhythmia. Therefore, CoQ-deficiency-related genes should be considered in the molecular autopsy of SUD.

## Electronic supplementary material

Below is the link to the electronic supplementary material.

Supplementary Material 1

Supplementary Material 2

Supplementary Material 3

## Data Availability

Pathogenic or likely pathogenic variants have been submitted to the Leiden Open Variation Database (http://databases.lovd.nl/shared/diseases). All data will be provided upon request to the first or corresponding authors.
