# Supplementary figures and images for: Coenzyme Q deficiency may predispose to sudden unexplained death via an increased risk of cardiac arrhythmia
Source: Int J Legal Med. 2024 Jun 7;138(6):2239–48. doi: 10.1007/s00414-024-03265-5 (PMC11490525; doi:10.1007/s00414-024-03265-5)

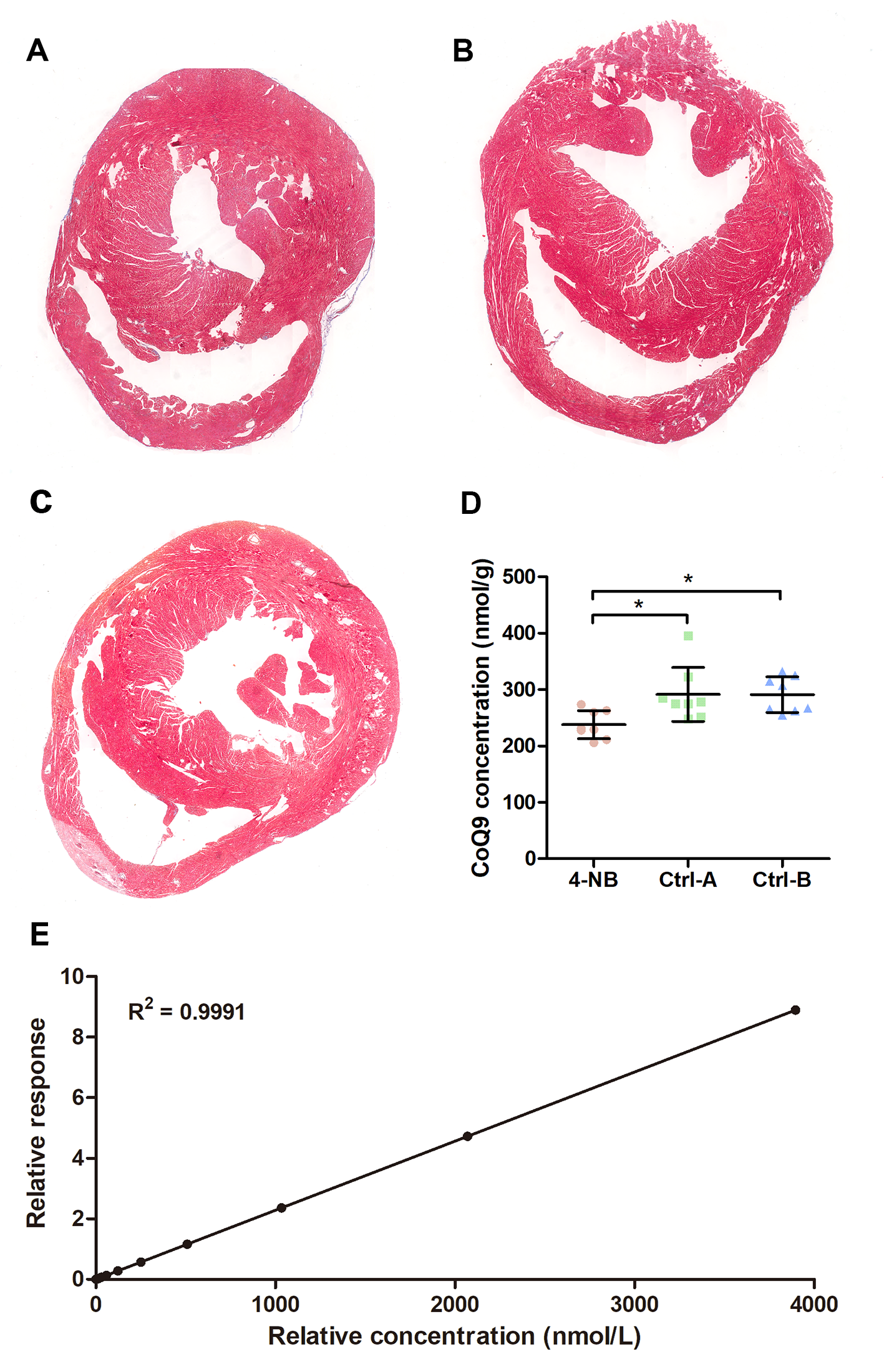

Supplement: Supplementary file 2 — Supplementary Material 2 [file 414_2024_3265_MOESM2_ESM.tif]
